# Mast Cells and Mas-related G Protein-coupled Receptor X2: Itching for Novel Pathophysiological Insights to Clinical Relevance

**DOI:** 10.1007/s11882-024-01183-5

**Published:** 2024-11-25

**Authors:** Mariana Castells, Michael Madden, Carole A. Oskeritzian

**Affiliations:** 1 Division of Allergy and Clinical Immunology, Brigham and Women’s Hospital, Harvard Medical School, Smith Building, Room 626D, 1 Jimmy Fund Way, Boston, MA 02115 USA; 2 Department of Pathology, Microbiology, and Immunology, University of South Carolina School of Medicine, Building 2, Room C10, 6439 Garners Ferry Road, Columbia, SC 29209 USA

**Keywords:** Mast cell, Drug hypersensitivity, Allergy, FceRI, MRGPRX2, Non-IgE

## Abstract

**Purpose of Review:**

Clinical interest in non-IgE activation of mast cells has been growing since the description of the human MRGPRX2 receptor. Its participation in many allergic and inflammatory conditions such as non histaminergic itch, urticaria, asthma and drug hypersensitivity has been growing. We present here an updated review of its structure, expression and biology to help understand conditions and diseases attributed to its activation and/or overpexression and the search for agonists and antagonists of clinical utility.

**Recent Findings:**

The description of patients presenting anaphylaxis when exposed to one or multiple MRGPRX2 agonists such as general anesthetics, antibiotics, opiods and other agents has provided evidence of potential heterogeneity in humans.

**Summary:**

This review provides the most recent developments into the receptor structure, tissue expression and signaling pathways including the potential enhancement of IgE-mediated mast cell activation. New insight into its agonists and antagonists is described and future developments to adress its modulations.

## Introduction

Mast cells are the most ancients cells of the immune system and have been identified in Urochordates (sea squirts) which are recorded to exist for the last 650 million years. Mast cells are found in most living organisms from fish, amphibians, reptiles, birds, and all mammals [1] and are strategically located in humans in the skin and mucosal membranes to sample microbes and environmental agents. Mast cells communicate with neighboring cells and tissues through activating and inhibitory receptors and through soluble factors. Granule mediators such as histamine and proteases, and membrane derived prostaglandins and leukotrienes can act locally and can also travel in the blood stream and activate target receptors in all organs [2]. While the high affinity IgE receptor FceRI is expressed in all mast cells and considered the most important activating receptor, and it has become apparent in the last 15 years that G-coupled protein non IgE binding receptors are clinically relevant. MRGPRX2 is the first G-coupled receptor capable of activating mast cells by drugs bearing THIQ motifs such as quinolones and general anesthetics [3], in addition to basic compounds. Expression of this receptor is tissue specific and a pathogenic role has been attributed in diseases such as drug allergy, asthma and urticaria as well as immune surveillance. Patients with cutaneous mastocytosis have increased expression of MRGPRX2 in skin lesions [4] and patients with chronic urticaria have overall increased skin expression [5]. Immune cells recruitment and an antimicrobial role has been shown through MRGPRX2 [6] and rhinovirus induced increase in beta defensins production in bronchial epithelial cells has been shown to activated MRGPRX2 and mediate asthma exacerbations [7]. Non -histaminergic itch has been attributed to MRGPRX2 [8]. Hymenoptera and drug induced anaphylaxis in patients with negative skin testing has now been attributed to altered or increased expression of mast cells MRGPRX2 [9]. To provide new insight in the understanding of this receptor we reviewed its structure, expression and functions and describe agonists and antagonists with clinical relevance.

### Structure

**Fig. 1:**
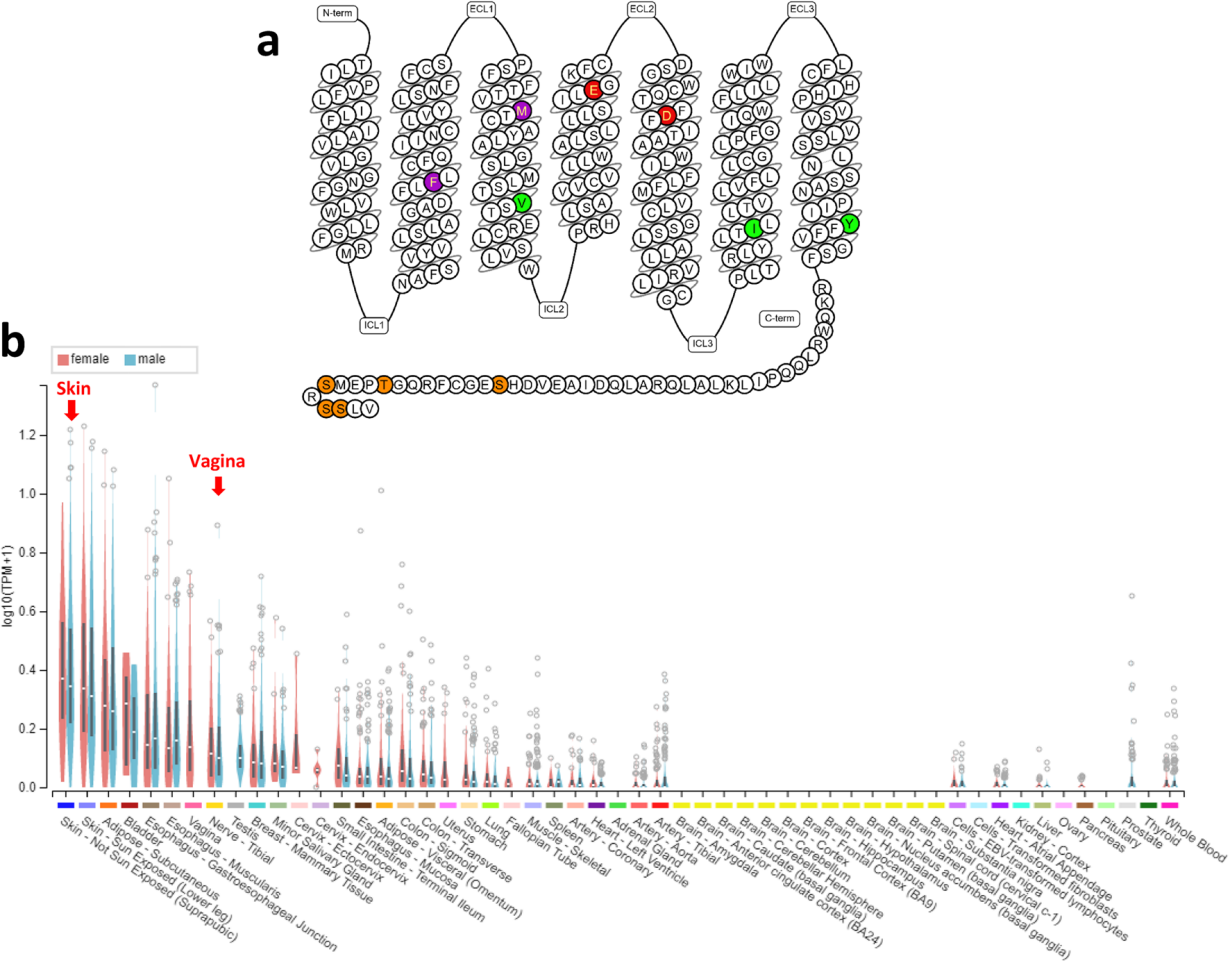
Snake diagram of MRGPRX2 (**a**). Sex-specific bulk tissue-level gene expression of MRGPRX2 (gtexportal.org/home/gene/ENSG00000183695) (**b**)

Since the cloning of a family of G-protein-coupled receptors (GPCR) named Mas-related GPCR after the first discovered member, Mas [10] the Mrgpr family has expanded to 50 genes and pseudogenes in mice and 18 in humans. The demonstration that a number of these receptors conveyed itch sensation was enabled by the development of mice genetically ablated for some of these genes [11]. Remaining “orphaned” for nearly sixty years after the initial observation that compound 48/80 was a potent mast cell activator [12], MRGPRX2 (X2) was demonstrated as the receptor for compound 48/80 predominantly expressed on human mast cells [13]. X2 was next identified as responsible for acute, non-IgE mediated reactions through binding to many US Food and Drug Administration-approved drugs [3]. A better understanding of this receptor’s binding ability to so many ligands was achieved once the 3-D crystal structure of X2 was fully solved in 2021. Indeed, two concurrent reports unveiled the high-resolution liganded structure of X2 using electron cryo-microscopy [14, 15]. Importantly, both studies elucidated how X2 may ligate a broad range of structurally diverse cationic molecules through a shallow binding pocket featuring a negatively charged sub pocket [16] and a hydrophobic sub pocket [14, 15] (Fig. 1). In addition, MRGPRX2 was missing several canonical trigger motifs present in other GPCRs, including the classical ‘toggle switch’ tryptophan (replaced by a glycine) implicated in the initial conformational changes classically associated with receptor activation [15]. This substitution forces ligation at a position distant from the conventionally described agonist binding sites for GPCRs. Moreover, the conserved disulfide bond between Transmembrane (TM) domain 3 and extracellular loop 2 (ECL2) present in family A GPCRs was absent in MRGPRX2. Instead, predicted to be found in all the human MRGPR family receptors, a TM4-TM5 inter-helix disulfide bond occurs that was demonstrated to be necessary to preserve X2 signaling integrity [15]. The lack of TM3-ECL2 disulfide bond enables ECL2 to flip to the top of TM4 and TM5, yielding an aberrantly wide-open ligand-binding surface thus providing an explanation for X2 binding to a large array of ligands. Further, Yang et al. identified a peptide motif common to several peptidergic allergens, allowing binding to X2 [14]. Using homology modeling of human X2, docking simulations and functional assays (calcium mobilization), fluoroquinolone-binding sites and key residues of X2, namely M109 and F78, were recently identified whose missense mutation mutations would exacerbate responsiveness to *e.g.*, ciprofloxacin, raising the possibility for diverse ligands to interact with different amino acids near the predicted ligand-binding pocket [17, 18]. It is noteworthy that no structural information is currently available for the inactive states of X2 and other MRGPR, thus how the transition from inactive to active states occurs remains to be elucidated. The structural resolution of X2 has been critical to our understanding of ligand binding, for drug development of specific receptor antagonists and agonists [19, 20] and future therapies [15, 21].

### Expression

MRGPRX2 is expressed in sensory neurons, mast cells and in keratinocytes [10, 22–24]. MRGPRX2 mRNA was detected in adipose tissue, esophagus, urinary bladder, and lungs with the highest levels found in skin [25] (Fig. 1). Transcripts were not detected in kidney, liver, ovary, spleen, or pancreas [26] (https://www.ncbi.nlm.nih.gov/gene/117194). Its highest expression remains on human skin mast cells, strongly supporting X2’s critical roles in local responses such as injection-site reactions. Babina et al. recently demonstrated that X2 served as the exclusive opiate receptor expressed by skin mast cells [27]. Importantly, some X2 ligands, including codeine [27], trigger its internalization, thus a refractory state of desensitization not only to codeine but also to other X2 ligands. Increased expression of X2 on skin mast cells was observed in patients with chronic spontaneous urticaria [5] who displayed exacerbated skin reactivity upon intradermal injections of X2 agonists such as substance P [28]. Elevation of X2 expression was also observed in patients with allergic contact dermatitis and asthma [29] (Fig. 3). However, whether X2 elevation may be the sole culprit of systemic drug hypersensitivity reactions remains controversial [30]. A case report described a patient with systemic mastocytosis who suffered multiple episodes of anaphylaxis to Hymenoptera venom and ciprofloxacin [9], both X2 stimulators [3], suggesting X2-mediated systemic mast cell activation. It is noteworthy that systemic effects may engage other effector cells, including basophils (whose X2 expression remains controversial) and neurons. Systemic perioperative reactions may result from the administration of several X2-stimulating drugs. Variants of X2 have been described that may influence X2 expression levels on mast cells and other cells [31]. A GPCR database identified over 100 missense SNPs [32]. Responsiveness to MRGPRX2 ligands may vary not only because of genetic polymorphisms but also be due to distinct receptor binding sites, differences in MRGPRX2 signalosome, epigenetic modifications, post-transcriptional modifications, dynamics of surface expression and tissue environment.

### Signaling Profiles

**Fig. 2:**
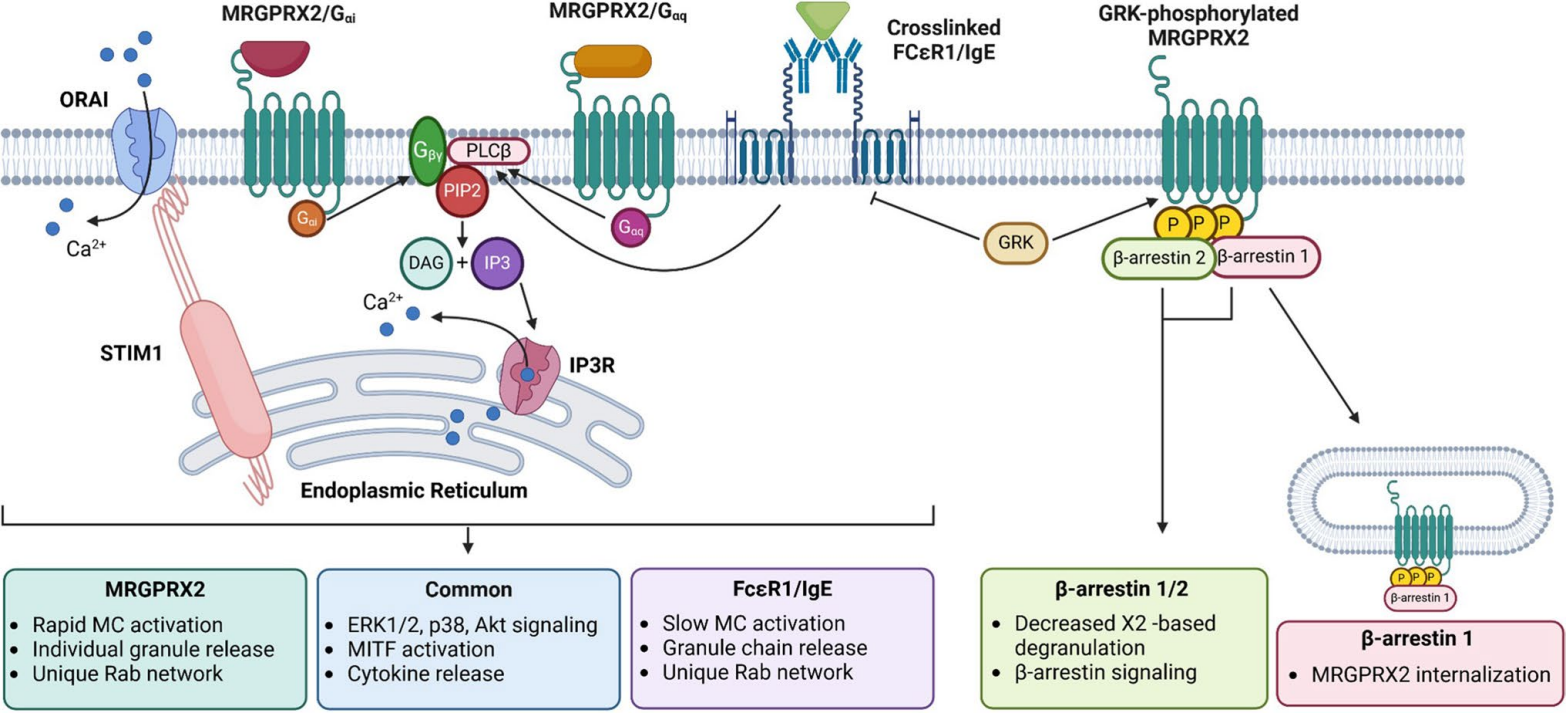
Comparison of MRGPRX2 and FCεR1/IgE signaling pathways

X2 activation by compound 48/80 engages both G proteins and b-arrestins [27] (Fig. 2). Despite controversy regarding the canonical MRGPRX2 signaling pathways [31], Cao et al. established that MRGPRX2 may effectively couple to nearly all G-protein families [15], with robust coupling at both Gq- and Gi-family α-subunits [33]. Activated Gi protein may release Gbg that, in turn, recruit the phospholipase C-b to the membrane and further promote Gq-mediated signaling [34]. Naturally occurring X2 mutations may alter accessibility to the ligand-binding pocket [15], binding affinity, downstream signaling, including G protein coupling. X2 activation engages multiple signaling pathways, including increased phosphorylation of phospholipase C-g (PLC-g), extracellular signal-regulated protein kinase 1/2 (ERK1/2), and Akt [35]. In addition, an early-phase and sustained calcium flux response also contributes to X2 signaling, through store-operated Ca^2+^ entry (SOCE) enabled by the Ca^2+^ sensor stromal interaction molecule 1 (STIM1) [36]. Silencing of Orai1, Orai2 or Orai3 (CRAC/orai) decreased X2/substance P-induced calcium mobilization, degranulation and cytokine/chemokine production [19].

b-arrestin-coupled signaling conveys receptor desensitization, internalization and signaling termination [37–39] and b-arrestin-1 and b-arrestin-2 display 78% sequence homology and contribute to significant regulation of GPCR functions, including X2 with G-protein-coupled receptor kinases (GRK) [40]. Differential regulation of FceRI- *vs*. Mrgprb2 [mouse ortholog of X2]-mediated MC responses has been shown by GRK2, inhibiting IgE-triggered degranulation but enhancing b2/X2-dependent MC activation [41, 42]. Binding of b-arrestins to GRK-phosphorylated receptors promotes their internalization without degradation [43]. In human skin MC, b-arrestin-1 is required for agonist-induced X2 internalization [44]. The structure of the ligand may bias the receptor to preferentially activate one over the other G protein, whereas a balanced (unbiased) agonist would activate all G proteins [45]. A balanced X2 ligand can induce MC degranulation as well as receptor internalization such as compound 48/80, substance P and codeine [27, 46]. Roy et al. recently reported that X2 may participate in rosacea-like inflammation, highlighting a pro-inflammatory role for b-arrestin-2 [47]. An open resource, PRESTO-Tango, uses a b-arrestin recruitment The TANGO (transcriptional activation following arrestin translocation) assay as outcome to identify new X2 agonists [48].

The transcriptional program essential for mast cell development, functions and maintenance encompasses several transcription factors (TFs), including microphthalmia-associated transcription factor (MITF), GATA binding protein 2 (GATA-2), and STAT-5 [49]and a recent study highlighted a prominent role of X2 activation to phosphorylate and activate MITF [50]. This occurs through the nuclear translocation of Lysyl t-RNA synthetase, an event also reported downstream of FceRI crosslinking [51], indicating that the MITF pathway is an integral part of X2 signaling that is shared with FceRI.

### Mechanisms of Activation

**Fig. 3:**
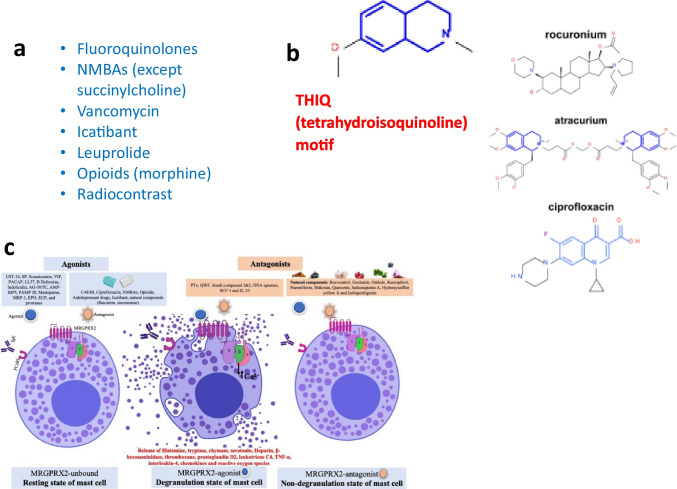
MRGPRX2 Agonists and Antagonists (**a**, **c**). THIQ motif identified in several MRGPRX2- activating drugs (**b**)

The tetrahydroisoquinoline (THIQ) motif, one of the drug binding sites of X2, is found in small compound drugs, including neuromuscular blocking agents (NMBAs, used during general anesthesia such as cisatracurium and rocuronium [52]) and fluoroquinolone antibiotics (ciprofloxacin, moxifloxacin, levofloxacin, and ofloxacin) [3] (Fig. 3) The THIQ motif is also present in drugs used in the treatment of diseases ranging from advanced Parkinson disease (apomorphine), cancer (*e.g.*, trabectedin), to parasitic diseases (praziquantel). The benzylisoquinoline alkaloids (BIAs) are a family of THIQ natural products that include morphine, codeine, and their analogs. It is well known that opioids, particularly morphine, trigger a rapid but transient elevation of plasma histamine [53, 54].

Recent studies suggested the possibility of shared pathways between X2- and IgE-mediated activation of mast cells, with regards to cytokine release [55]. Similar kinases were shown to be engaged downstream of both receptors, including ERK1/2, and to a lesser extent, p38 and protein kinase B [35]. The rapid signaling activation upon X2 ligation as compared to the canonical IgE/FceRI route, has been thought to be relevant to the involvement of X2 in immediate hypersensitivity reactions. The inverted kinetics of activation reported for these two receptors [X2 rapid, FceRI slow] could be explained by contrasted patterns of degranulation; whereas FceRI crosslinking triggers the fusion of intracytoplasmic granules with each other forming a chain of granules, X2 activation results in the release of individual granules [56]. The process of granule discharge requires phosphoinositide 3-kinase downstream of both receptors [35]. Important to granule exocytosis are Rab GTPases that control vesicular trafficking [57]. A recent study identified a network of Rabs regulating X2 responses, several of which inhibited secretion that were shared with FceRI, while others were uniquely impacting each receptor [58]. In the absence of specific mediators released via one receptor and not the other and additive effects on mast cell exocytosis is possible, broadening the clinical relevance of X2 activation in urticaria, mastocytosis and atopic dermatitis [59].

### Agonists and Antagonists

Because of the established relevance of X2 in the sensation of itch and pain, designing drugs to regulate X2 activation is clinically relevant. Among the few reported X2 antagonists, compound EP262 was the first-in-class once-daily oral selective small molecule antagonist to be granted initiation of a Phase 1 study by the US FDA in January of 2023, a multicenter, randomized, placebo-controlled Phase 2 for CSU and a Phase 2a study for atopic dermatitis. Several approaches are undertaken to design and test novel X2 antagonists [60, 61]. Agonists of MRGPR neurons were reported to inhibit MRGPRB2-dependent activation of mast cells in mice [62], a novel approach for the regulation of pseudoallergic reactions. In silico strategies may complement the conventional biological screening of drug libraries for the design of specific drugs targeting X2.

Kumar et al. identified the natural compound genistein as therapeutically useful to the development of functional X2 antagonists [63]. The same group utilized an integrated computational approach encompassing structure-aided screening and drug design, docking, synthetic, as well as preclinical experimental pharmacology to develop small molecule X2 antagonist derived from genistein [60]. These newly developed small molecules dose-dependently inhibited in vitro (human cell line) and ex vivo (mouse peritoneal cells) mast activation, as measured by b-hexosaminidase release, calcium flux, chemokine and lipid mediator production, and receptor activation quantified by b-arrestin recruitment and showed promising inhibitory effects using in vivo mouse models of acute inflammation and systemic anaphylaxis. Additional studies pertaining to structure optimization are required to increase potency of lead compounds.

## Conclusions and Future Directions

While more functions of X2 are uncovered, the search for pharmacological agents to inhibit or agonize its functions may not use standard knockout mouse models since b2, the mouse ortholog of X2, and X2 display large differences in ligand action concentrations [3]. Furthermore, there are many more MRGPR receptor family members in mice compared to humans (with low amino-acid identity between species), increasing the risk for compensatory mechanisms for lost-of-function in knockout mice. Humanized mice will be key to the development of preclinical models more relevant to the study of human X2, complemented by ex vivo approaches utilizing human primary mast cells, rather than cell lines. Most importantly data bases of patients reactive to quinolones and general anesthetics can be used to identify populations at risk and genotypic studies may uncover variants conferring high risk for reactions. Biobanks with blood and tissues sections from these patients will allow for tissue transcriptomics and identification of pathogenic variants. Patients with mastocytosis reactive to X2 activating drugs are currently being identify [64] and guidelines for the avoidance, desensitization or use of pre-medications should be established.

## Data Availability

No datasets were generated or analysed during the current study.
